# Expanded spectral domain-OCT findings in the early detection of hydroxychloroquine retinopathy and changes following drug cessation

**DOI:** 10.1186/s40942-016-0042-y

**Published:** 2016-07-18

**Authors:** David R. Lally, Jeffrey S. Heier, Caroline Baumal, Andre J. Witkin, Steven Maler, Chirag P. Shah, Elias Reichel, Nadia K. Waheed, Igor Bussel, Adam Rogers, Jay S. Duker

**Affiliations:** 1 Vitreoretinal Service, Departments of Ophthalmology, New England Eye Center, Tufts University School of Medicine, 260 Tremont Street, Boston, MA 02116 USA; 2 Vitreoretinal Service, Ophthalmic Consultants of Boston, Boston, MA USA

## Abstract

**Purpose:**

To report expanded SD-OCT findings of HCQ retinopathy that may assist the clinician in earlier diagnosis. To characterize structural changes of HCQ retinopathy with SD-OCT after drug cessation.

**Methods:**

Setting: Private practice and academic institution. Patient Population: Patients at New England Eye Center and Ophthalmic Consultants of Boston in Boston, MA diagnosed with HCQ retinopathy and followed after drug cessation. Retrospective clinical data review by the Boston Image Reading Center. Main Outcome Measures: SD-OCT findings suggestive of HCQ retinopathy before parafoveal ellipsoid disruption. Change in SD-OCT morphological appearance and retinal thickness of each of the nine subfields corresponding to the Early Treatment of Diabetic Retinopathy Study areas.

**Results:**

Thirty eyes with HCQ retinopathy were followed with SD-OCT after drug cessation. Findings before disruption of the parafoveal EZ included parafoveal outer nuclear layer (ONL) thinning, disruption of the parafoveal interdigitation zone, and reduced reflectivity of the parafoveal EZ. In early toxicity, 75 % developed progression after drug cessation, including disruption of the parafoveal EZ and retinal pigment epithelium and thinning of the ONL. Eyes with obvious toxicity had greater inferior outer ring thinning 12 months after drug cessation compared to early toxicity (p = 0.002, 95 % CI −2 to −8 μm). In obvious toxicity, the nasal inner subfield showed more thinning than the temporal inner subfield at 12 months after drug cessation (p = 0.018, 95 % CI −1 to −8 μm).

**Conclusions:**

Once HCQ retinopathy is diagnosed and the medication is discontinued, structural retinal changes commonly occur.

## Background

Hydroxychloroquine (HCQ) has been used for therapy of rheumatologic disorders since the 1950s. Ocular toxicity associated with HCQ use was initially described in the 1960s [1, 2]. The incidence of HCQ retinopathy is estimated at 1 % after consumption of HCQ for 5 years [3]. It is marked by paracentral and central scotoma and decreased color vision. Risk factors predisposing to the development of HCQ retinopathy include a daily dose >6.5 mg/kg, cumulative dose >1000 g, duration of use over 5 years, elderly age, kidney or liver dysfunction, or preexisting maculopathy [4]. HCQ retinopathy is rare considering the widespread utilization of this drug; however, the retinopathy has potential to produce irreversible visual loss [1].

There is limited data regarding structural and functional changes of HCQ retinopathy after drug cessation [5–7]. Studies from the 1960s and 1970s evaluated individuals taking the more toxic, related drug chloroquine, but were limited to visual acuity, visual fields, and fundus examination to assess for progression of retinopathy. In addition, these studies typically evaluated patients with advanced disease who had already developed a Bull’s eye fundus appearance. In the present day, the less toxic HCQ is used instead of chloroquine. The American Academy of Ophthalmology revised the screening guidelines in 2011 to include spectral domain-optical coherence tomography (SD-OCT), fundus autofluorescence (FAF), and multifocal ERG (mfERG) when available to assess the macula in patients taking HCQ [4]. The new guidelines were designed for earlier detection of HCQ retinopathy before funduscopic changes become apparent. Studies reviewing mfERG and FAF after drug cessation have demonstrated both progression and improvement in retinal function in moderate to severe toxicity [8–11]. It is unclear whether less severe cases of retinopathy progress after HCQ cessation.

SD-OCT is a highly sensitive and reproducible imaging modality commonly used in clinical practice. SD-OCT is capable of detecting characteristic macular changes of HCQ toxicity. These typical macular abnormalities include loss of the parafoveal ellipsoid zone (EZ), parafoveal thinning of the outer nuclear layer (ONL) and inner plexiform layer (IPL), the “flying saucer” sign, and peripapillary nerve fiber layer thinning [12–14]. Few reports have examined progression of changes in SD-OCT appearance after drug cessation. Marmor et al. [11] reported that SD-OCT appearance in patients with early HCQ toxicity, defined as patchy parafoveal visual field defects, did not change after drug cessation. However, moderate cases, defined as a 50–100 % parafoveal ring of damage on visual field testing, did demonstrate progressive parafoveal thinning. Kellner et al. [15] showed long-term follow-up of eyes with moderate to severe chloroquine/HCQ toxicity resulting in cystoid macular edema and epiretinal membrane formation. However, no changes were observed in early toxicity.

This study examines longitudinal SD-OCT scans after cessation of HCQ and stratifies eyes based on severity of retinopathy to evaluate whether this plays a role in structural changes. An independent reading center was used to limit observational bias in interpreting the SD-OCT scans. Furthermore, SD-OCT findings are identified before clear parafoveal ellipsoid zone (EZ) disruption becomes apparent, which may enable earlier diagnosis of HCQ retinopathy.

## Methods

Data collection was obtained following approval from the Internal Review Board (IRB) of Tufts Medical Center, and was in compliance with the Health Insurance Portability and Accountability Act. Research tenets were followed in accordance with the Declaration of Helsinki.

A retrospective, consecutive case series of eyes with HCQ retinopathy that were followed after drug cessation was conducted at New England Eye Center, Boston, Massachusetts and Ophthalmic Consultants of Boston, Boston, Massachusetts. The study interval was from January 1, 2006 to July 12, 2013. Eyes were included if they were diagnosed with HCQ retinopathy and had SD-OCT imaging at the time of drug cessation and on follow-up exam. In addition, eyes were included only if the follow-up SD-OCTs were performed on the same machine—either Cirrus HD-OCT (Carl Zeiss Meditec, Dublin, CA) or Heidelberg Spectralis HRA + OCT (Heidelberg Engineering, Germany).

HCQ retinopathy was diagnosed by detecting abnormalities attributable to HCQ on clinical examination, Humphrey 10-2 or 24-2 visual fields, SD-OCT, and/or FAF when available. Of note, all eyes in this study had visual field defects consistent with HCQ toxicity. Multifocal ERG was performed in only 8 eyes, and in these eyes, toxicity was confirmed with visual field defects and SD-OCT findings. Therefore, mfERG was not included in the analysis. Eyes were excluded if there was no clinical examination on record coincident with the time of HCQ cessation, if the follow up OCT was performed on a different OCT machine than at the time of drug cessation, or if time-domain OCT was performed. Ocular symptoms, best-available visual acuity, macular appearance, and imaging modalities (OCT, FAF) available at the time of HCQ retinopathy diagnosis and after HCQ cessation were recorded. The cumulative HCQ dose was calculated.

The first primary outcome measure was the morphological change in 1-line horizontal OCT scans from the time of discontinuing HCQ to the most recent examination. Vertical OCT scans were not routinely ordered by the treating physician and therefore unable to be included in analysis. If there were SD-OCT images available that demonstrated findings that preceded disruption of the parafoveal EZ, these findings were recorded and analyzed by the reading center. The second primary outcome measure was the change in retinal thickness in each of the 9 Early Treatment of Diabetic Retinopathy Study (ETDRS) OCT sub-fields at 12 months after drug cessation.

The SD-OCT scans were evaluated by two masked, trained readers from the Boston Image Reading Center (BIRC, Boston, MA). The readers were provided raw data from the cube scans, which were qualitatively assessed for alterations in the normal morphological retinal features. Readers analyzed SD-OCT images from the time of drug cessation and most recent follow up exam, and assessed whether any interval changes had occurred. After careful deliberation, common features which were most consistently recorded were decreased reflectivity of the ellipsoid zone (EZ), disruption of the EZ, disruption of the interdigitation zone (IZ), disruption of the retinal pigment epithelium (RPE), disruption of the external limiting membrane (ELM), and thinning of the outer nuclear layer (ONL). These characteristics were evaluated to be present or absent in both the foveal region (central 1500 μm) and parafoveal region (0–2000 μm from the border of the foveal region on either side). The findings were considered true findings only if both readers agreed on the finding (i.e. hyporeflective, RPE atrophy, etc.) and the location of the finding (i.e. fovea, nasal parafovea, temporal parafovea, or both nasal and temporal parafovea). In no instances was there an opposite finding on either side of the fovea, such as hyperreflectivity on the nasal side and hyporeflectivity on the temporal side.

**Fig. 1:**
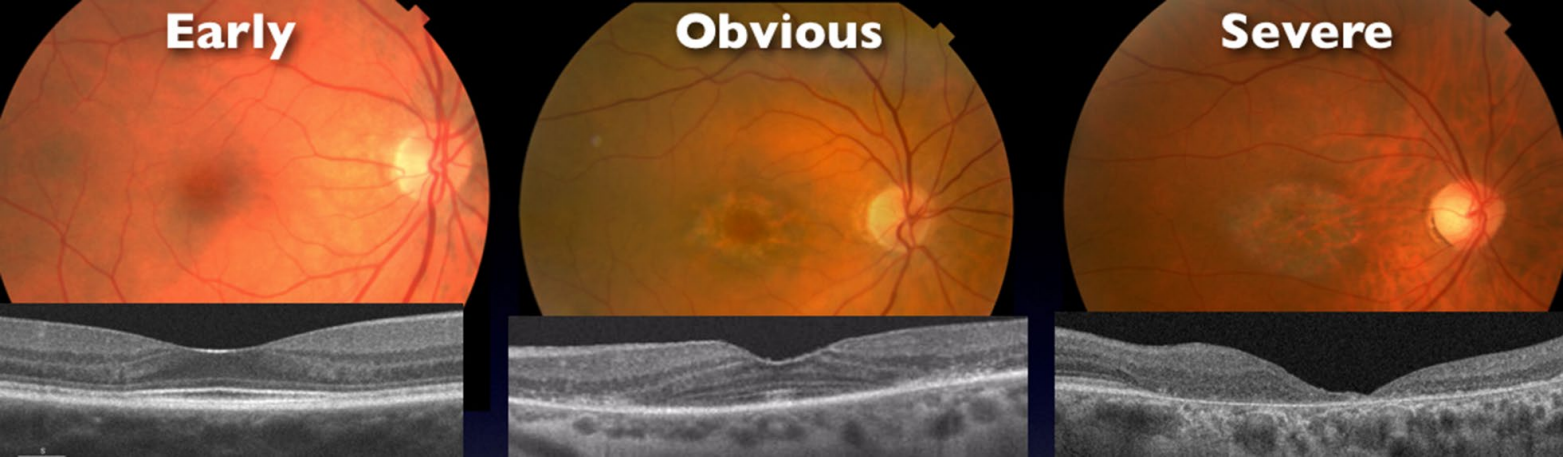
Fundus photograph and SD-OCT example of each group. Cohort divided into three groups based on BIRC’s grading of ellipsoid zone at the time of drug cessation. *Early*—no disruption of parafoveal or foveal EZ; *Obvious*—disruption of parafoveal EZ with intact foveal EZ; *Severe*—disruption of both foveal and parafoveal EZ

 For data analysis, the primary cohort was divided into three groups based on BIRC’s grading of the 1-line horizontal OCT scan through the fovea at the time of drug cessation (Fig. 1). The purpose of the division was to separate early, obvious, and severe cases of toxicity, as the development of new screening modalities such as SD-OCT has allowed for detection of earlier cases of HCQ toxicity. The *Early* group consisted of eyes presenting without clear disruption of both the foveal and parafoveal ellipsoid zone (EZ) as judged by BIRC’s interpretation of the SD-OCT at the time of drug cessation. Fundus examination of these eyes showed early macular findings, or none at all, at the time of diagnosis. The *Obvious* group consisted of eyes presenting with an intact foveal EZ but a clearly disrupted parafoveal EZ on either one or both sides of the fovea as judged by the BIRC graders. These eyes clinically manifested the classic parafoveal RPE changes. The *Severe* group consisted of eyes presenting with clear foveal EZ disruption with associated parafoveal EZ disruption. These eyes had poor visual acuity at presentation due to foveal and parafoveal outer retinal atrophy.

Retinal thickness measurements of each of the nine subfields corresponding to the Early Treatment of Diabetic Retinopathy Study (ETDRS) areas were examined. ETDRS SD-OCT thickness measurements consist of nine separate regions, which are defined by three concentric rings centered on the fovea with diameters of 1, 3, and 6 mm, respectively. The two outer rings are divided into quadrants by two intersecting orthogonal lines. Only eyes that underwent SD-OCT imaging at baseline and follow-up with either Cirrus HD-OCT (Carl Zeiss Meditec, Dublin, CA) or Heidelberg Spectralis HRA + OCT (Heidelberg Engineering) were included in this analysis. Eyes that had baseline Stratus OCT (Carl Zeiss Meditec, Dublin, CA) were excluded.

Statistical analysis was performed using GraphPad (GraphPad Software, Inc., La Jolla, CA USA). We used an unpaired *t* test to compare the changes in retinal thickness between the groups, while a paired t-test was used to compare the changes within a group. 95 % confidence intervals were calculated for any statistically significant changes observed as defined as p < 0.05.

## Results

Thirty-six patients were identified with HCQ retinopathy. Thirteen patients were excluded because an OCT was not performed at the time of diagnosis. Eight patients were excluded for having a time domain-OCT at the time of drug cessation. Thirty eyes of 15 female patients met inclusion criteria and were followed by repeated examinations after drug cessation. Clinical features of each patient are outlined in Table 1 and in each of the three groups in Table 2. All patients were Caucasian. Half of the eyes were emmetropic, and half were ametropic (range −6.0 to +6.0 diopters). None of the patients had kidney or liver dysfunction.

**Table 1:** Clinical summary of patients

Patient/sex/age	HCQ cumulative dose (g)	Body mass index	HCQ indication	Follow-up after HCQ cessation (months)	HCQ cessation VA	Most recent VA	Symptoms at HCQ cessation	Symptoms at follow-up	Progression after HCQ cessation?
E1/F/57	730	^a^	Psoriatic arthritis	13	OD:20/20 OS:20/20	OD:20/20 OS:20/20	None	None	Yes (OCT, HVF)
E2/F/74	890	^a^	SLE	13	OD:20/30 OS:20/40	OD:20/40 OS:20/40	Decreased near vision	Decreased near vision	Yes (OCT)
E3/F/69	584	51	RA	56	OD:20/25 OS:20/25	OD:20/30 OS:20/30	None	None	Yes (OCT, HCF)
E4/F/61	438	26	RA	12	OD:20/25 OS:20/25	OD:20/25 OS:20/25	Floaters	None	No
E5/F/65	949	17	RA	42	OD:20/20 OS:20/20	OD:20/20 OS:20/20	Floaters	None	Yes (OCT, HVF)
O1/F/48	2210	^a^	SLE	18	OD:20/30 OS:20/40	OD:20/30 OS:20/30	None	None	Yes (OCT)
O2/F/65	4380	24	RA	36	OD:20/20 OS:20/20	OD:20/20 OS:20/20	Floaters	Floaters	Yes (OCT)
O3/F/62	1022	24	RA	42	OD:20/50 OS:20/60	OD:20/40 OS:20/50	Blurry vision	Blurry vision	No
O4/F/63	2482	^a^	SLE	36	OD:20/25 OS:20/25	OD:20/25 OS:20/25	Floaters	Flashes	Yes (OCT)
O5/F/69	1550	^a^	SLE	12	OD:20/25 OS:20/25	OD:20/25 OS:20/25	Glare	Glare, floaters	No
O6/F/67	1933	24	SLE	12	OD:20/25 OS:20/25	OD:20/25 OS:20/25	None	None	Yes (OCT)
O7/F/68	1048	26	RA	36	OD:20/20 OS:20/30	OD:20/30 OS:20/40	None	Decreased near vision	Yes (OCT, FP)
O8/F/75	2628	24	RA	12	OD:20/25 OS:20/25	OD:20/25 OS:20/25	None	Decreased near vision	Yes (OCT)
S1/F/63	1030	23	RA	36	OD:20/400 OS:20/100	OD:20/400 OS:20/150	Decreased central vision	Decreased central vision	Yes (OCT)
S2/F/76	1679	19	RA	14	OD:20/150 OS:20/50	OD:20/100 OS:20/100	Blurry vision	Blurry vision	No

E2 left eye was included in *Severe* group analysis. E3 right eye was included in *Obvious* group analysis

^a^Information unavailable

**Table 2:** Baseline characteristics of each group

	Early	Obvious	Severe
Age (years)	63 (57–74)	65 (48–75)	74 (63–76)
Cumulative dose (g)	730 (438–949)	1933 (584–4380)	1030 (890–1679)
Baseline vision	20/20–20/40	20/20–20/60	20/100–20/400
Follow-up (months)	13 (12–56)	36 (12–56)	14 (13–36)

Median (range) reported for age, cumulative dose, and follow-up

SD-OCT data was available for all 30 eyes at the time of drug cessation and follow up examinations. The *Early* group comprised of eight eyes of 5 patients. The *Obvious* group comprised of 17 eyes of 9 patients. The *Severe* group comprised of 5 eyes of three patients. The right eye of patient E2 qualified for the *Early* group and the left eye for the *Severe* group. The right eye of patient E3 qualified for the *Obvious* group and the left eye for the *Early* group. All qualitative observations had a strong inter-observer correlation between the two observers from the reading center.

### SD-OCT at time of drug cessation

**Fig. 2:**
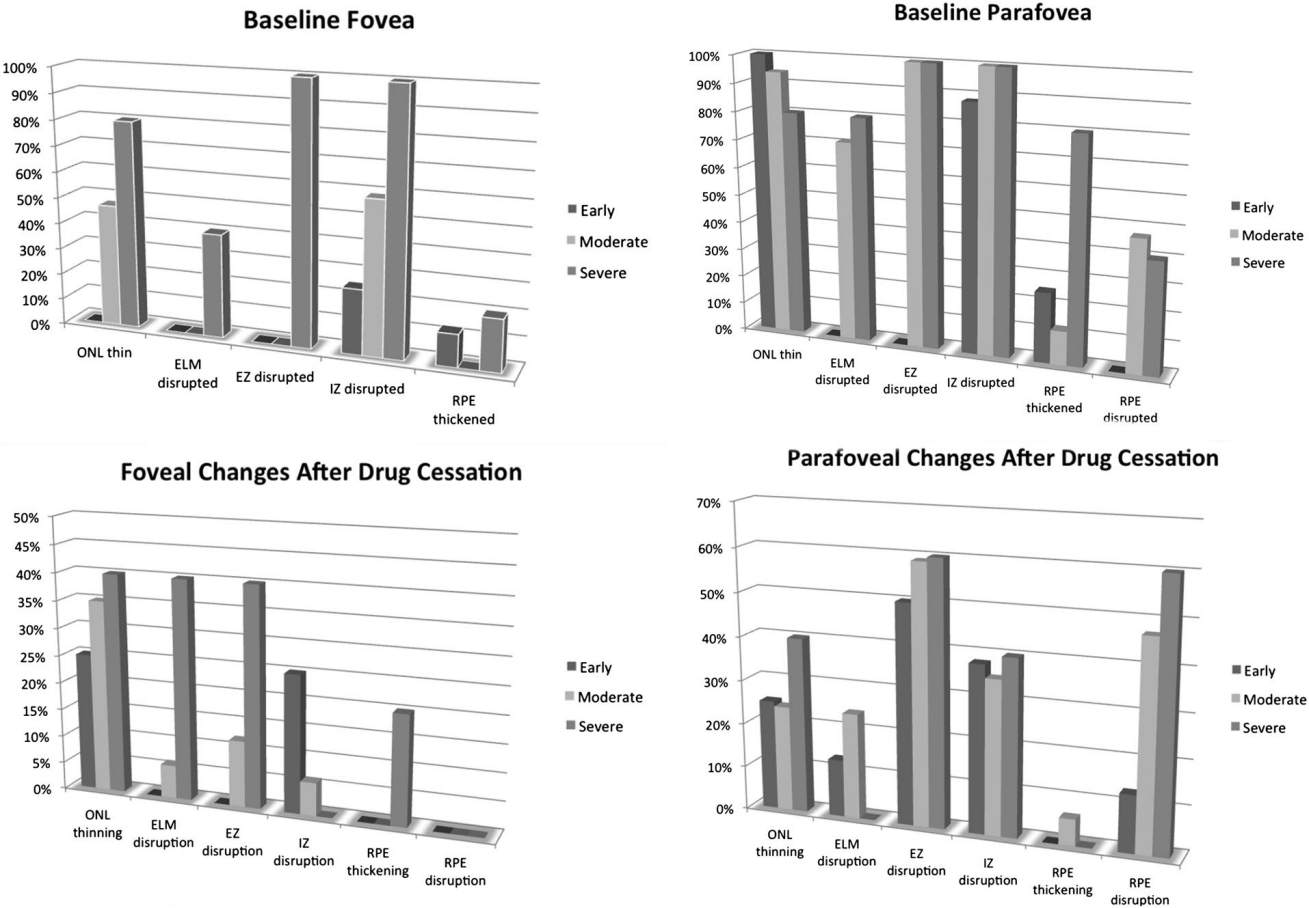
(*Top row*) reading center’s grading of SD-OCTs at time of HCQ discontinuation in fovea and parafovea. (*Bottom row*) reading center’s grading of interval changes in SD-OCT appearance of most recent follow-up exam compared to time of HCQ cessation. If thinning, disruption, or thickening was present at the time of drug cessation and an interval change was noted, the change was progressive or worsening thinning, disruption, or thickening

Morphological appearance of 1-line horizontal SD-OCT scans in each of the groups at the time of drug cessation is shown in Fig. 2. In the fovea, 25 % of the *Early* eyes and 59 % of the *Obvious* eyes had clear disruption of the interdigitation zone (IZ) in the absence of ellipsoid zone disruption, suggesting that IZ disruption may precede EZ disruption. By our definition of the three groups, no patients in the *Early* or *Obvious* group had foveal disruption of the EZ while all patients in the *Severe* group did. In the *Severe* group, foveal external limiting membrane (ELM) disruption was observed in 40 %, while this finding was not present in the other two groups. Foveal retinal pigment epithelium (RPE) disruption was not observed at baseline in any group. Foveal thinning of the outer nuclear layer (ONL) was not present at baseline in any group.

**Fig. 3:**
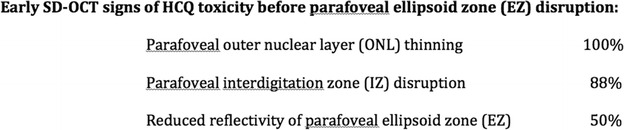
SD-OCT findings of early toxicity preceding the development of parafoveal EZ disruption

**Fig. 4:**
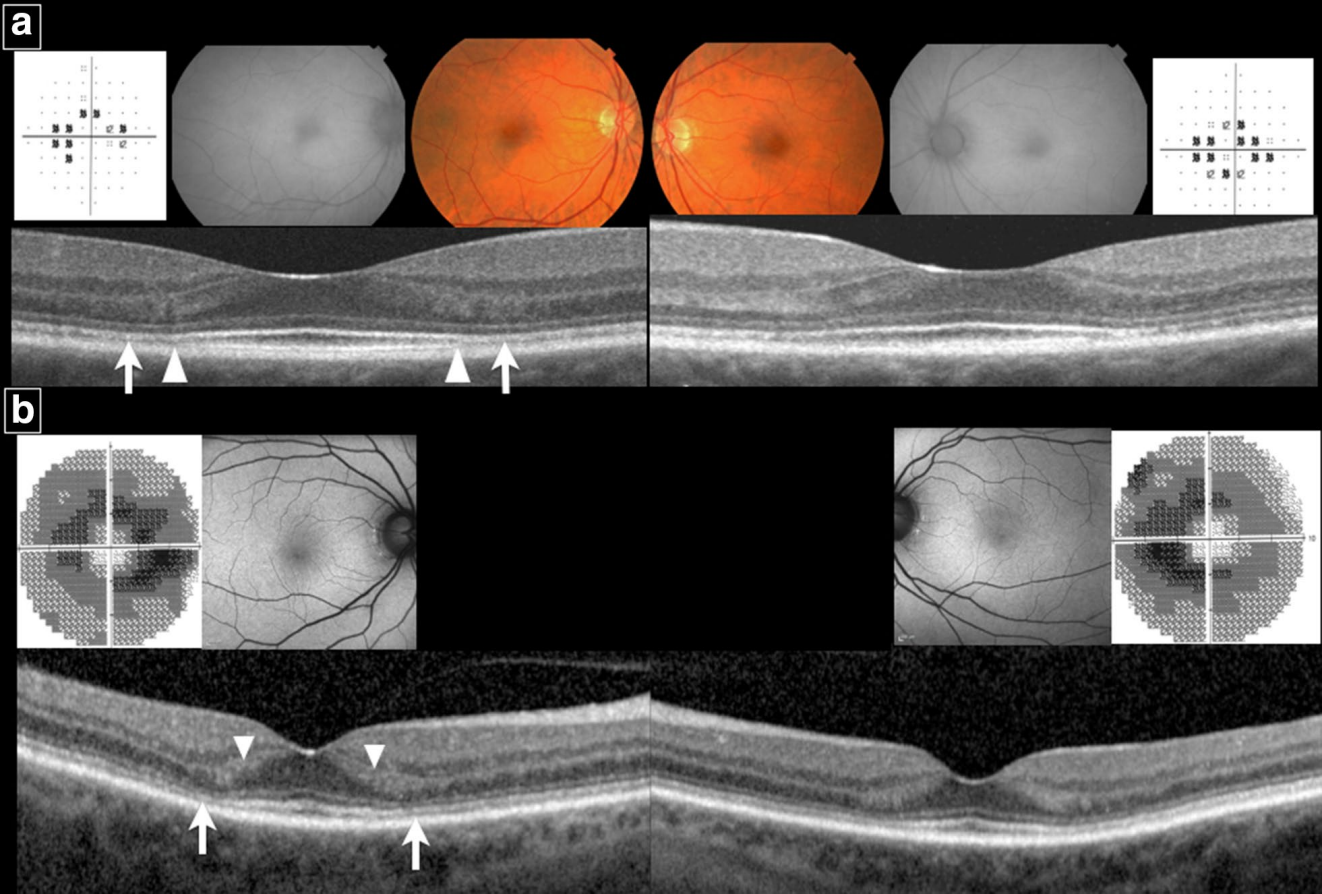
Examples of two *Early* patients. **a** E1, fundus photograph shows mild parafoveal RPE changes in each eye with unremarkable FAF. HVF 10-2 shows incomplete parafoveal ring scotoma in *right* eye and parafoveal ring scotoma in *left* eye. SD-OCT of each eye shows no clear disruption of parafoveal EZ. However, reduced reflectivity of the parafoveal EZ (*arrow*) on both sides of the fovea is observed and enhances the appearance of the foveal EZ. Abrupt discontinuation of the parafoveal interdigitation zone (IZ) (*arrowhead*) is also observed on both sides of the fovea. **b** E4, Fundus autoflourescence is unremarkable. HVF 10-2 shows incomplete parafoveal ring scotomas in each eye. No clear disruption of the parafoveal EZ is seen on SD-OCT. However, the reflectivity of the parafoveal EZ is diminished (*arrow*) and enhances the appearance of the foveal EZ. There is also thinning of the parafoveal outer nuclear layer causing broadening of reflectivity of the parafoveal Henle’s fiber layer (*arrowhead*) on both sides of the fovea

In the parafoveal region, early SD-OCT findings were frequently detected in *Early* eyes in the absence of clear parafoveal EZ disruption (Fig. 3). Outer nuclear layer (ONL) thinning was present in 100 % and observed as a focal indentation in the parafoveal ONL when compared to peripheral ONL thickness. Disruption of the parafoveal interdigitation zone (IZ) was recorded in 88 % and reduced reflectivity of the parafoveal EZ band in the absence of definitive disruption was observed in 50 %. The reduced reflectivity of the parafoveal EZ enhanced the appearance of the foveal EZ, creating the illusion of increased prominence or hyperreflectivity of the foveal EZ (Fig. 4). Parafoveal RPE disruption was not detected in *Early* eyes, but was notable in 47 % of *Obvious* eyes and 40 % of *Severe* eyes.

### Progression of SD-OCT changes after drug cessation

**Fig. 5:**
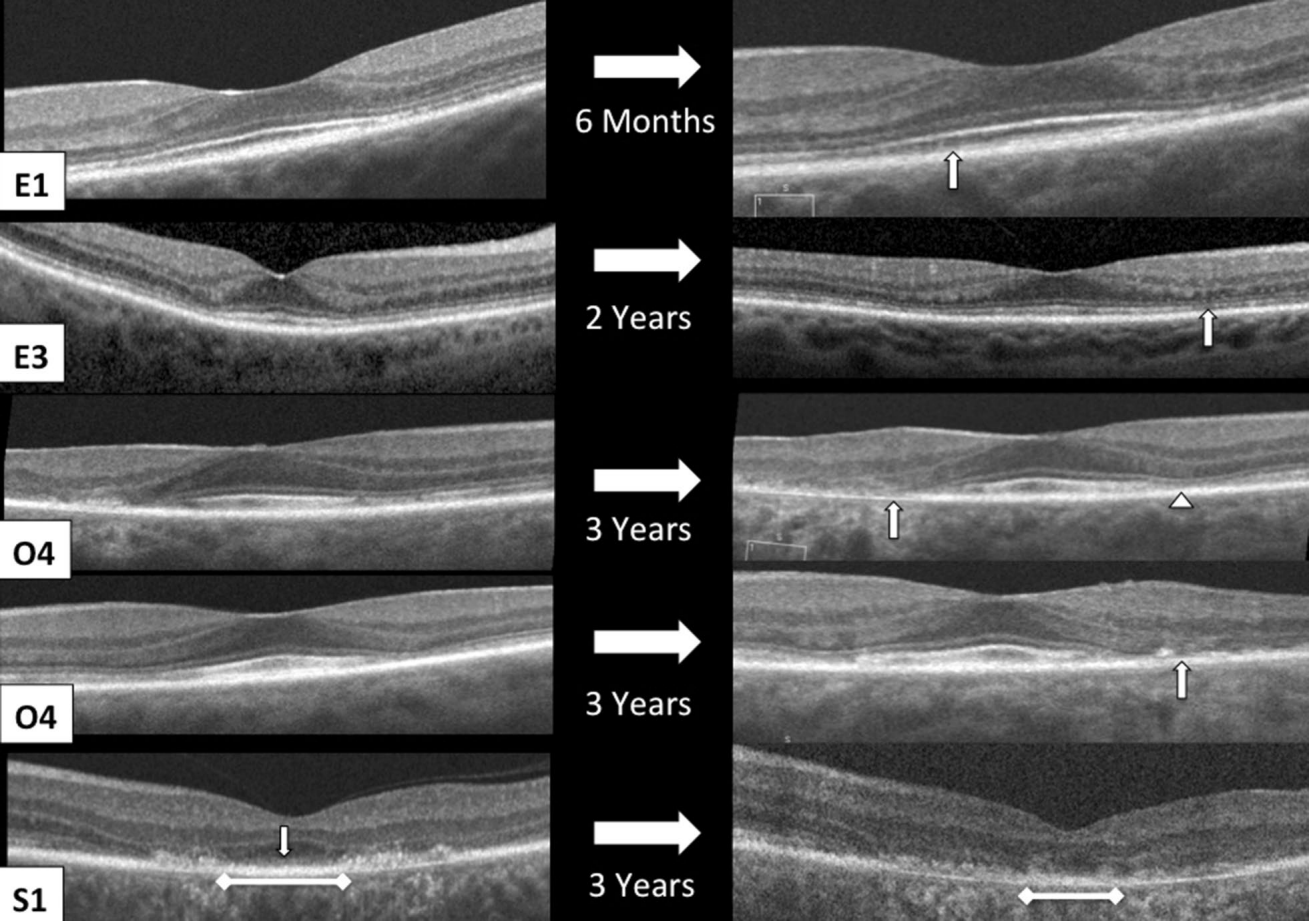
Examples of changes in SD-OCT appearance from the time of diagnosis of HCQ retinopathy (*left column*) to after drug cessation (*right column*). E1, *left* eye, shows progressive interdigitation zone disruption (*arrow*). E4, *right* eye, shows development of parafoveal EZ disruption (*arrow*). O4, *right* eye, shows progressive RPE atrophy of the temporal parafovea (*arrow*) and progressive EZ disruption of the nasal parafovea (*arrowhead*). O4, *left* eye, shows progressive disruption of the ELM, EZ, and RPE in the temporal parafovea (*arrow*). S1, *left* eye, shows progressive loss of the foveal ELM (*arrow*), foveal RPE migration into outer retina, and disruption with shortening of foveal RPE (ruler)

**Fig. 6:**
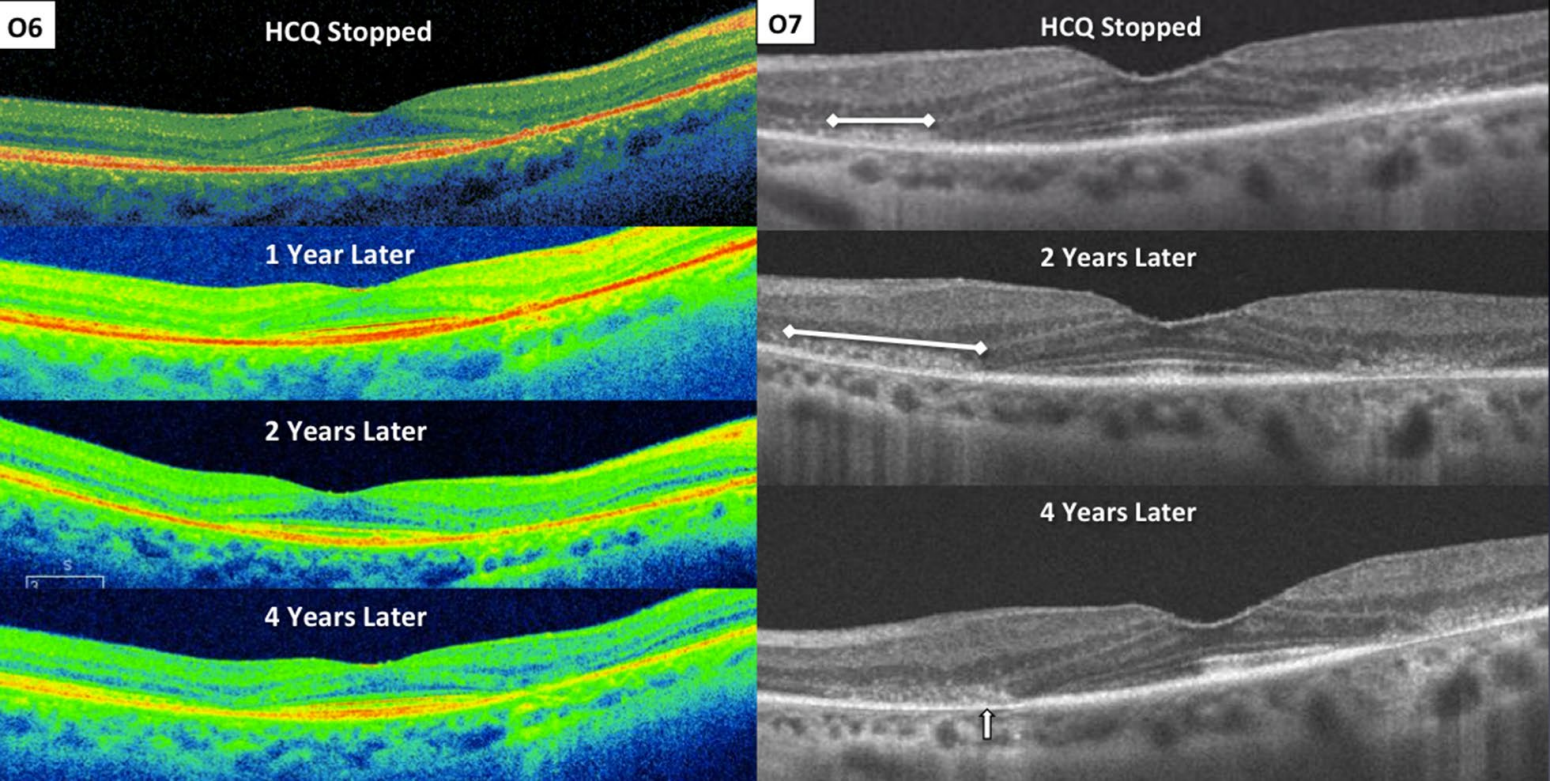
SD-OCT changes 4 years after HCQ cessation. O6, *right* eye, shows development of parafoveal RPE atrophy that becomes evident 2 years after drug cessation and progresses at 4 year follow up. O7, *left* eye, shows progressive parafoveal RPE atrophy (*arrow*) with broadening of RPE migration into outer retina in the nasal parafovea (ruler)

Morphological SD-OCT changes in each group after drug cessation is shown in Fig. 2. An interval change in SD-OCT appearance suggesting structural progression of toxicity was observed in 75 % *Early*, 71 % *Obvious,* and 100 % of *Severe* eyes. In the *Early* group, 50 % developed clear parafoveal EZ disruption after drug cessation with a median follow-up of 13 months (range 12–77 months; Fig. 5). Increased parafoveal EZ disruption was observed in 60 % of the *Obvious* and *Severe* eyes. Increased parafoveal RPE disruption was detected in 13 % *Early* eyes, 47 % *Obvious* eyes, and 60 % *Severe* eyes. There was no significant difference in the frequency or severity of temporal parafovea changes compared to the nasal parafovea. Foveally, the most common change seen on SD-OCT after drug cessation was progressive ONL thinning in 25 % *Early*, 35 % *Obvious*, and 40 % *Severe* eyes. Progressive foveal RPE disruption was not detected. The latest time point in which progression was observed was progressive parafoveal RPE atrophy continuing for 4 years after drug cessation (Fig. 6). Finally, a reduction in disruption or improvement in SD-OCT appearance was not observed.

### Macular thickness changes

**Fig. 7:**
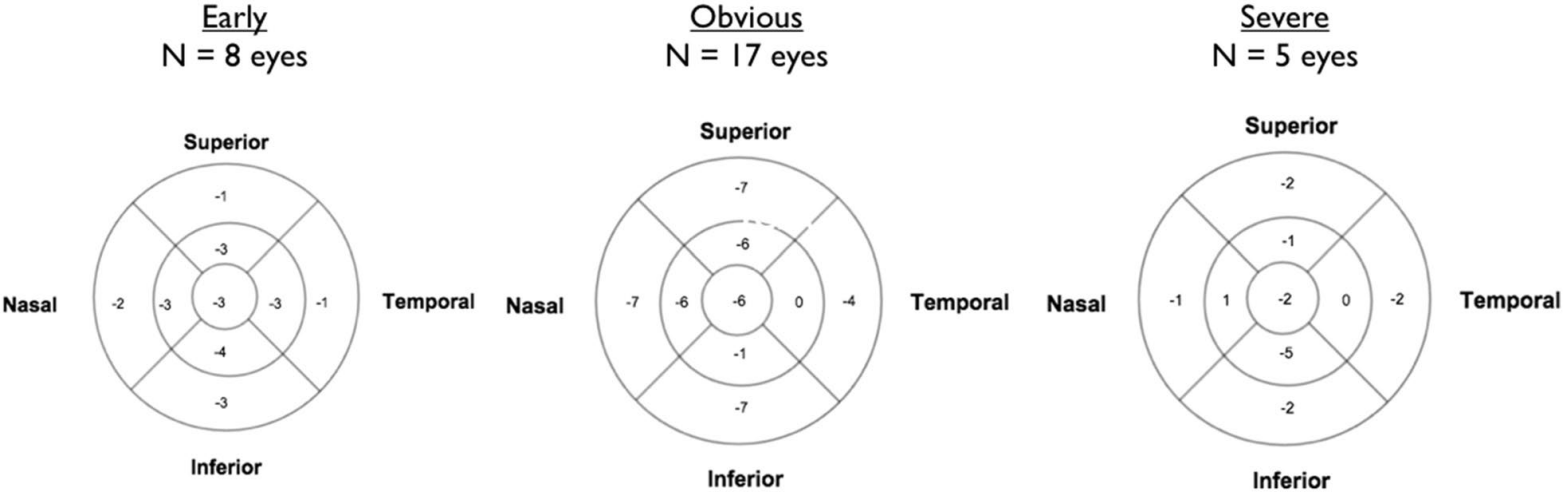
Median change in thickness in each ETDRS subfield at 12 months after HCQ cessation

Figure 7 illustrates the change between HCQ cessation and 12 months after cessation in each of the 9 ETDRS SD-OCT subfields. Decreases in central macular thicknesses were 3, 6, and 2 μm in the *Early*, *Obvious*, and *Severe* groups, respectively. The largest reduction in ETDRS map region thickness was 7 μm in the superior, nasal, and inferior outer rings of the *Obvious* group. When comparing thickness changes in the *Obvious* group to the *Early* group, a significant difference was found in the inferior outer ring (p = 0.002, 95 % CI −2 to −8 μm). No differences between the groups were found in the other subfields. When comparing different subfield thickness changes within an individual group, no differences were observed within the *Early* or *Severe* groups. However in the *Obvious* group, the nasal inner subfield showed more thinning than the temporal inner subfield (p = 0.018, 95 % CI −1 to −8 μm; Table 3).

**Table 3:** P values for the comparison of ETDRS subfield thickness changes for *Obvious* group

	Early	Obvious	Severe
Nasal versus temporal inner	1.000	*0.018* *CI* = − *8 to* −*1*	0.660
Superior versus inferior inner	0.756	0.226	0.658
Nasal versus temporal outer	1.000	0.285	0.155
Superior versus inferior outer	0.626	0.291	0.264

Italic value indicates significance of p value (p < 0.05)

*CI* 95 % confidence interval in micrometers

**Fig. 8:**
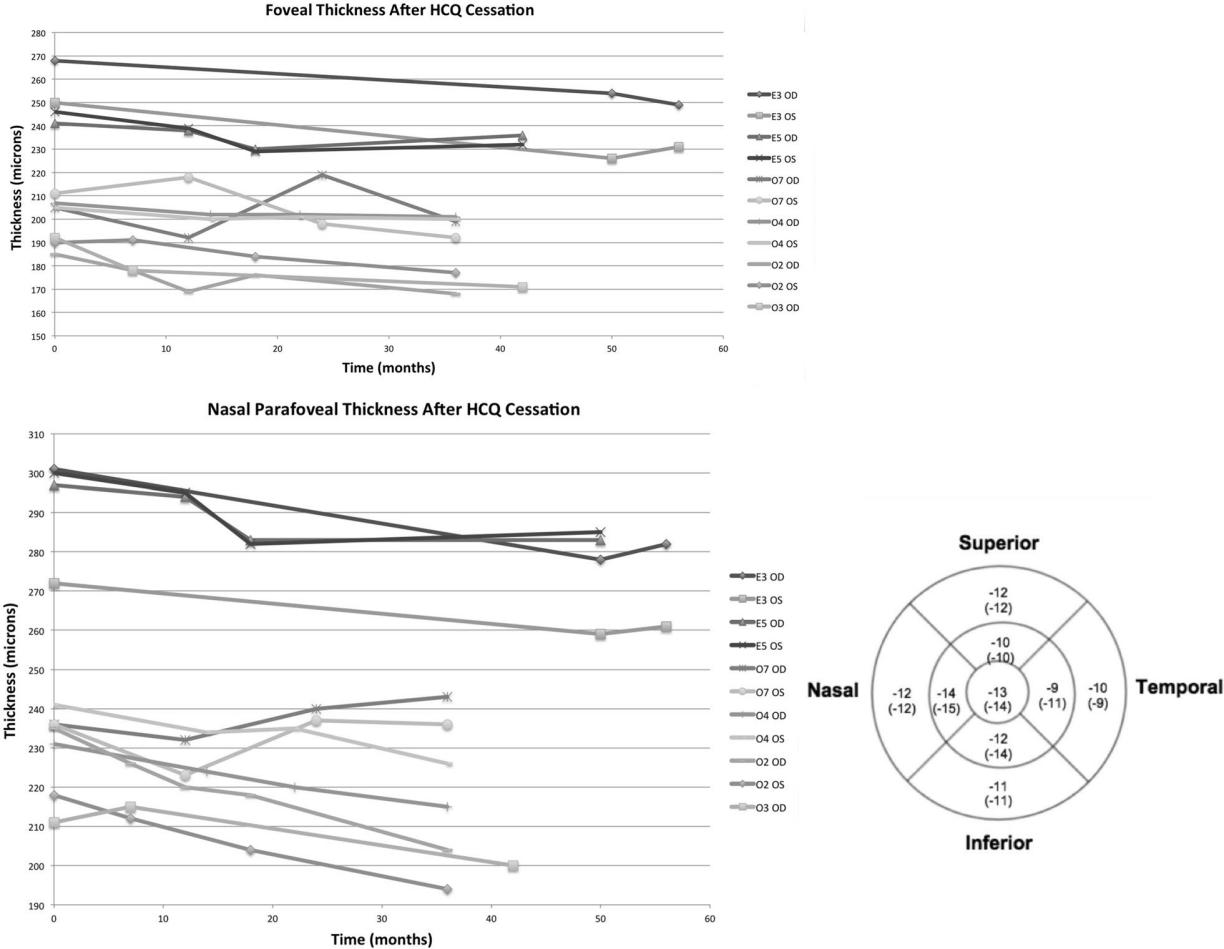
Subanalysis for eyes with at least 3 years of follow-up after drug cessation (E3, E5, O2, O3 OD, O4, O7). (*Top left*) Subfoveal thickness change after drug cessation. (*Bottom left*) Example of nasal inner ring ETDRS subfield thickness change after drug cessation. (*Bottom right*) Mean change (Median change) in thickness in each ETDRS subfield

A subanalysis of eyes with at least 3 years of follow-up after drug cessation (N = 11 eyes; patients E3, E5, O2, O3 OD, O4, O7; O3 OS excluded because of poor quality image at 42 month follow-up visit; S1 excluded because of widespread atrophy) was performed (Fig. 8). A progressive decline in retinal thickness beyond 1 year was observed in 62 % of eyes. Median subfoveal thickness reduction was 14 μm. The largest decline in thickness was observed in the nasal inner ring (15 μm). A difference in thickness change was not detected between nasal and temporal outer or inner rings (p = 0.50; p = 0.11, respectively) or superior and inferior outer and inner rings (p = 0.60; p = 0.65, respectively). In the *Early* patients, *E3* developed thinning at 50 months compared to baseline, but had no follow-up visits in between. Therefore, it is uncertain at what time point the thinning occurred. Patient E5 showed thinning for the first 18 months after drug cessation but then the thickness increased between months 18 and 42.

## Discussion

By using masked, trained SD-OCT graders from the Boston Image Reading Center (BIRC), we determined that structural retinal changes were common after HCQ cessation, occurring in 73 % of all eyes included in this study at a mean follow up of 26 months. All changes were consistent with progression of toxicity and may continue beyond 1 year most commonly as thinning of the parafoveal RPE (Fig. 6). In the *Early* group, 75 % had evidence of structural progression after drug cessation, including disruption of the parafoveal EZ and RPE and thinning of the ONL (Table 1). These eyes developed structural progression despite presenting with subtle changes on SD-OCT at the time of HCQ cessation. Three of the five *Early* patients developed worsening visual field defects in conjunction with structural changes. Importantly however, visual acuity and symptoms in these patients did not change significantly.

The disruption of the parafoveal ellipsoid zone is well-reported in HCQ toxicity, and clinicians commonly look for this finding in clinical practice [4, 11–13]. In this report, we describe three additional, clinically useful early SD-OCT signs of HCQ retinal toxicity that may precede disruption of the parafoveal ellipsoid zone (Fig. 3). We recommend looking carefully for these early findings to assist in the earliest detection of retinopathy. If an abnormality is detected, the authors recommend carefully checking the visual field searching for paracentral defects, as all *Early* eyes in this series had paracentral visual field defects at the time of drug cessation. This fact has been reported previously by Marmor and Melles [16] who described eyes with HCQ retinopathy in which the SD-OCT images were interpreted as normal but had paracentral visual field defects consistent with HCQ toxicity. Our review of these published images reveals that 8 of the 10 eyes displayed reduced reflectivity of the parafoveal EZ creating the appearance of a prominent foveal EZ band, suggesting that those eyes may have had reduced reflectivity of the parafoveal ellipsoid zone, as found in many of the *Early* eyes in our study. A criticism of this finding that we recognize is that the finding may be too subtle to be assuredly detectable in clinical practice. Often a ‘normal’ SD-OCT can have variance in scan brightness affecting EZ reflectivity. However, in these cases, the entire EZ band, including the foveal and parafoveal EZ, should have homogenous or near homogenous reflectivity. In our study, the masked graders were agreeable to the interpretation of EZ reflectivity, and therefore, we believe the reduced reflectivity of the parafoveal EZ is a real detectable SD-OCT finding in early HCQ toxicity. This sole finding should not be diagnostic of toxicity, but rather used to raise suspicion for toxicity and generate further investigation.

Disruption of the parafoveal interdigitation zone (IZ) was another common finding (88 %) preceding parafoveal EZ disruption. The interdigitation zone, also known as the cone outer segment tip (COST) line, is the bright reflective line located between the EZ and the retinal pigment epithelium and is visible as a continuous line in 95 % of normal subjects [17]. This finding has been reported in HCQ retinopathy two times previously, and appears to be another important but early finding in *Early* HCQ toxicity [18, 19].

Macular thickness changes in the *Early* and *Severe* groups were minimal at 12 months after drug cessation. Changes in the *Severe* group were presumed secondary to glial and RPE remodeling as these eyes had severe parafoveal and foveal outer retinal atrophy at the time of drug cessation. *Obvious* eyes had foveal thinning at 12 months similar to a previous report [11], and the macular thinning was not equal between the subfields. Greater thinning was detected in the nasal inner ring when compared to the temporal inner ring. This may be attributable to a thicker baseline nasal inner retina with more opportunity for thinning compared to the temporal inner retina. In addition, *Obvious* eyes developed greater thinning in the inferior outer ring as compared to *Early* eyes. This suggests an acceleration of thinning in the inferior outer ring as toxicity progresses. Interestingly, two other studies have noted the inferior macula may be the earliest affected quadrant in HCQ toxicity, but further studies are needed [18, 20]. Finally, this study included myopic eyes up to −6D. The relationship between axial length and macular thickness measurements needs to be further clarified because axial length has both been negatively correlated to macular thickness but also not observed [21, 22]. Therefore, it remains unclear whether macular thickness changes in myopic eyes are different from emmetropic eyes.

Retained HCQ in the RPE cell may be the underlying cause of progressive retinal thinning observed in *Obvious* eyes after 12 months drug cessation. After intravenous injection of chloroquine into a rabbit, high concentrations of chloroquine are found in the pigmented ocular tissues (RPE, choroid, iris) [23]. These levels remain high for over 2 months following a single administration of chloroquine. Albino rabbits, on the other hand, do not retain chloroquine in the uvea or RPE indicating that melanin pigment is responsible for the binding and trapping of chloroquine within the cell. Once inside the RPE cell, HCQ disrupts lysosomal function leading to lipofuscin accumulation and photoreceptor degeneration [24]. We suspect HCQ is retained in the RPE cell for a prolonged period of time after drug cessation and may lead to progressive RPE atrophy, retinal thinning, and worsening visual field defects as observed in this study.

We did not observe improvement in visual acuity or visual fields after drug cessation. However, a study by Mititelu et al. [25] reported two patients with improvement in visual acuity and visual fields after HCQ cessation. The authors also observed parafoveal EZ regeneration and suggested the presence of an intact ELM at the time of drug cessation as a positive predictor for EZ regeneration. In contrast, EZ regeneration was not observed in our study, but progressive EZ disruption was noted in over half. Further studies are needed to support these findings.

SD-OCT, in combination with Humphrey’s visual field 10-2, as well as fundus autofluorescence and multifocal ERG in select patients, is critical for the early detection of HCQ retinopathy. As shown by this study and by Marmor and Melles [16], cases of early HCQ toxicity that display visual field changes attributable to HCQ toxicity in the setting of a ‘normal SD-OCT’ may in fact have early changes on SD-OCT as described in this report. We did not, however, observe any cases of HCQ retinopathy in which the visual field was normal but where morphological SD-OCT changes were detected. Therefore, a reliable visual field may be the most sensitive test for detecting early toxicity. However, fields are often unreliable, and SD-OCT has value in assisting detection in these cases. SD-OCT can also be used to corroborate early visual field defects. Conversely, if a parafoveal field defect is detected, we recommend repeating the field before deeming the finding unreliable.

## Conclusions

SD-OCT can show signs of early HCQ retinopathy before unmistakable parafoveal EZ disruption develops, and these signs may assist in earlier detection of HCQ toxicity. Early detection is critical because both structural and functional progression may occur after the drug is discontinued.

## Abbreviations

SD-OCT: spectral domain-optical coherence tomography
FAF: fundus autofluorescence
mfERG: multifocal electroretinogram
HCQ: hydroxychloroquine
RPE: retinal pigment epithelium
EZ: ellipsoid zone
ONL: outer nuclear layer
